# Dr. Thomas S. Powell

**Published:** 1896-01

**Authors:** 


### DR. THOMAS S. POWELL.

The news of the death of Dr. Thomas S. Powell, of Atlanta, which occurred December 30th, will be learned with deep regret by hosts of friends throughout the State, and by hundreds of physicians who have been trained under his teaching. Though Dr. Powell's health had been slowly failing for two or three years, no one supposed that his end was so near. His fatal illness began on the 26th with symptoms of cerebral anemia and an unconsciousness which lasted more or less until his death.

Dr. Powell was born in Virginia in 1826, and here he received his early education. He graduated in medicine from the University of Pennsylvania in 1846. For a few years he practiced in Sparta, Ga., and in 1858 removed to Atlanta, where the remainder of his life has been spent in active professional work. For a few years he was Professor of Obstetrics in the Atlanta Medical College, but resigned the chair in 1866. He began the publication of the *Southern Medical Record* in 1873, and edited this journal with ability and success for several years. In 1879 Dr. Powell was the prime mover in the organization of the Southern Medical College, and since that date his life and best energies have been almost wholly identified with the fortunes of this institution. From the first he has been Professor of Obstetrics and also President of the Faculty. Dr. Powell was a clear thinker and a good teacher, who never tired of his work. He was possessed of a firm will power, an indomitable energy, high purposes and honest convictions. He was generous and loving toward his friends, and often spoke affectionately of those with whom he was associated in a teaching capacity. In the practice of his profession his charity clientele was large, and the service was always cheerfully given. "Dr. Powell had always enjoyed the confidence and esteem of his medical brethren; because he had not only been a skillful practitioner, but in spite of the seductions of a large and growing practice he never bartered his principles for gain or overlooked for an instant the moral ethics of the profession."

The funeral was conducted at Trinity Church, Atlanta, January 1st, in the presence of a large number of friends, members of the medical profession, the Faculty and students of the Southern Medical College. The remains were buried at Sparta, Ga.

